# Scaling up breastfeeding in England through the Becoming Breastfeeding Friendly initiative (BBF)

**DOI:** 10.1111/mcn.13443

**Published:** 2022-11-04

**Authors:** Rowena Merritt, Sally Kendall, Tamsyn Eida, Fiona Dykes, Rafael Pérez‐Escamilla

**Affiliations:** ^1^ Centre for Health Services Studies University of Kent Canterbury UK; ^2^ Maternal and Infant Nutrition and Nurture Unit (MAINN) University of Central Lancashire Preston UK; ^3^ Department of Social and Behavioural Sciences Yale University School of Public Health New Haven Connecticut USA

**Keywords:** baby friendly hospital initiative, breast milk, breastfeeding, breastfeeding promotion, breastfeeding support, developed countries

## Abstract

Breastfeeding is the most accessible and cost‐effective activity available to public health and has been shown to be one of the most effective preventive measures mothers can take to protect their children's health. Despite the well‐documented benefits, the UK has one of the lowest breastfeeding rates in the world. The Becoming Breastfeeding Friendly (BBF) toolkit was developed through highly structured technical and academic collaboration, led by Yale University. It provides an evidence‐based process to help countries assess their breastfeeding status and readiness to scale up, and identifies concrete measures countries can take to sustainably increase breastfeeding rates, based on data‐driven recommendations. BBF is grounded in the Breastfeeding Gear Model complex adaptive systems framework which is made up of eight simultaneous conditions that sustain breastfeeding. In 2018, a committee of multi‐agency stakeholders implemented the BBF process in England, collecting evidence to score the ‘gear’ components of England's breastfeeding environment against 54 benchmarks. The Training and Programme Delivery gear received the highest score, attributable to existing learning outcomes for health professionals and practitioners, peer supporters and specialist services, although there is a need for greater coordination and integration. The lowest scores were given for Promotion and Coordination, Goals and Monitoring due to the lack of a dedicated national strategy for breastfeeding and poor sharing of localised strategies and programmes. The process generated clear recommendations highlighting the need for more robust routine infant feeding data collection and reporting, and the necessity for strengthening leadership, monitoring and oversight to scale up and sustain breastfeeding.

## Key messages

- England's overall weighted Becoming Breastfeeding Friendly (BBF) Index score was 1.1 (range 0–3) representing a moderate scaling up environment (range 1.1–2.0). Five gears: *Political Will, Legislation and Policies, Funding and Resources; Training and Programme Delivery and Research and Evaluation* scored at a moderate gear strength, while the remaining three gears—*Advocacy, Promotion and Coordination Goals and Monitoring*—were weak.
- The BBF process for England highlighted substantial gaps in the current breastfeeding practice data and recommended that more robust routine, population‐level infant feeding data collection and reporting is initiated that goes beyond 6–8 weeks and up to 2 years.
- The process identified that the lack of a national infant feeding co‐ordinator role or national breastfeeding committee had resulted in no dedicated workplan and a lack of advocacy for breastfeeding programmes. The need for greater future coordination, strategic goal setting and consistent monitoring was recommended to strengthen the breastfeeding environment.

## Graphical abstract

England has one of the lowest breastfeeding rates in the world. We used the Becoming Breastfeeding Friendly evidence‐based process to assess England's breastfeeding status and readiness to scale up, identifying concrete measures to sustainably increase breastfeeding rates based on data‐driven recommendations. Here we discuss England's need for more robust routine infant feeding data collection and reporting, and the necessity for strengthening leadership, monitoring and oversight to scale up and sustain breastfeeding.

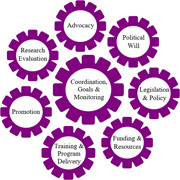

## 1 INTRODUCTION

Breastfeeding and the provision of human milk is well‐established as one of the most important human behaviours which benefits the child, the mother and society in general. It is the most accessible and cost‐effective activity available to public health, known to prevent a range of infectious and noncommunicable diseases, specifically gastro‐enteritis, childhood obesity, diabetes type 2 and maternal breast cancer (Renfrew et al., 2012; Victora et al., 2016).

The World Health Organization (WHO) recommends exclusive breastfeeding for the first 6 months of an infant's life, with continued breastfeeding up to 2 years of age or beyond, along with nutritionally adequate, safe and appropriate complementary foods (World Health Organization, 2003). However, breastfeeding practices are undermined by aggressive marketing of formula, negative societal attitudes, inadequate support from the health system, families and communities and within the workplace (Rollins et al., 2016). Global efforts to further improve exclusive breastfeeding rates have had limited success, in part because effective scaling‐up frameworks and roadmaps have not been sufficiently developed (Perez‐Escamilla et al., 2018).

The UK has one of the lowest breastfeeding rates in the world, with substantial variation across England. In 2014/2015, 74% of mothers started to breastfeed, falling to 44% breastfeeding at 6–8 weeks (NHS England, 2015), with considerable regional variation. The most recent publicly accessible ‘Fingertips’ public health data for England records ‘baby's first feed breastmilk’ (2018/2019) at 67% and ‘breastfeeding at 6–8 weeks’ (2020/2021) at 48% (Office for Health Improvement and Disparities, 2022), again varying by region. Only 1% of babies are exclusively breastfed in England until they are 6‐month‐old (McAndrew et al., 2012), with rates lowest among young, white women in routine or manual professions and who left education early, exacerbating health inequalities (Davies, 2014). Population level analysis and infant feeding data beyond 8 weeks is reliant on the most recently available nationally collected infant feeding data, the UK‐wide Infant Feeding Survey, from 2010; the survey has since been discontinued. There is therefore no current national data for exclusive breastfeeding at 6 months, or infant feeding data up to 2 years. There have also been a series of recent changes in how the routine data is collected and by whom. Data is currently reported as ‘experimental statistics’ to reflect the degree of change and the fact that the system remains under evaluation. At the current time, in England, the Maternity Services Data Set provides breastfeeding data directly after birth (whether a baby's first feed was breast milk [maternal or donor] or not breast milk), and data at 6–8 weeks is collected by local authorities and shared through the Community Services Data Set (CSDS) as part of the Maternity and Children's Data Set. There are inconsistencies in data collection between local authorities with a degree of inaccuracy and incompleteness therefore inherent in the data sets. The loss of the previous UK‐wide infant feeding survey has also meant that neither cross‐UK nor international comparisons can be made due to the different ways of collecting data and ways of defining breastfeeding.

### 1.1 The Becoming Breastfeeding Friendly (BBF) toolkit

The BBF toolkit was developed through highly structured technical and academic collaboration, led by Yale University and was piloted in Mexico and Ghana. In the short term, it provides an evidence‐based tool to guide countries in assessing their breastfeeding status, and their readiness to scale up. In the long term, it supports countries to identify the concrete measures they can take to sustainably increase breastfeeding rates, based on data‐driven recommendations (Hromi‐Fiedler et al., 2019; Pérez‐Escamilla et al., 2018).

**Figure 1:**
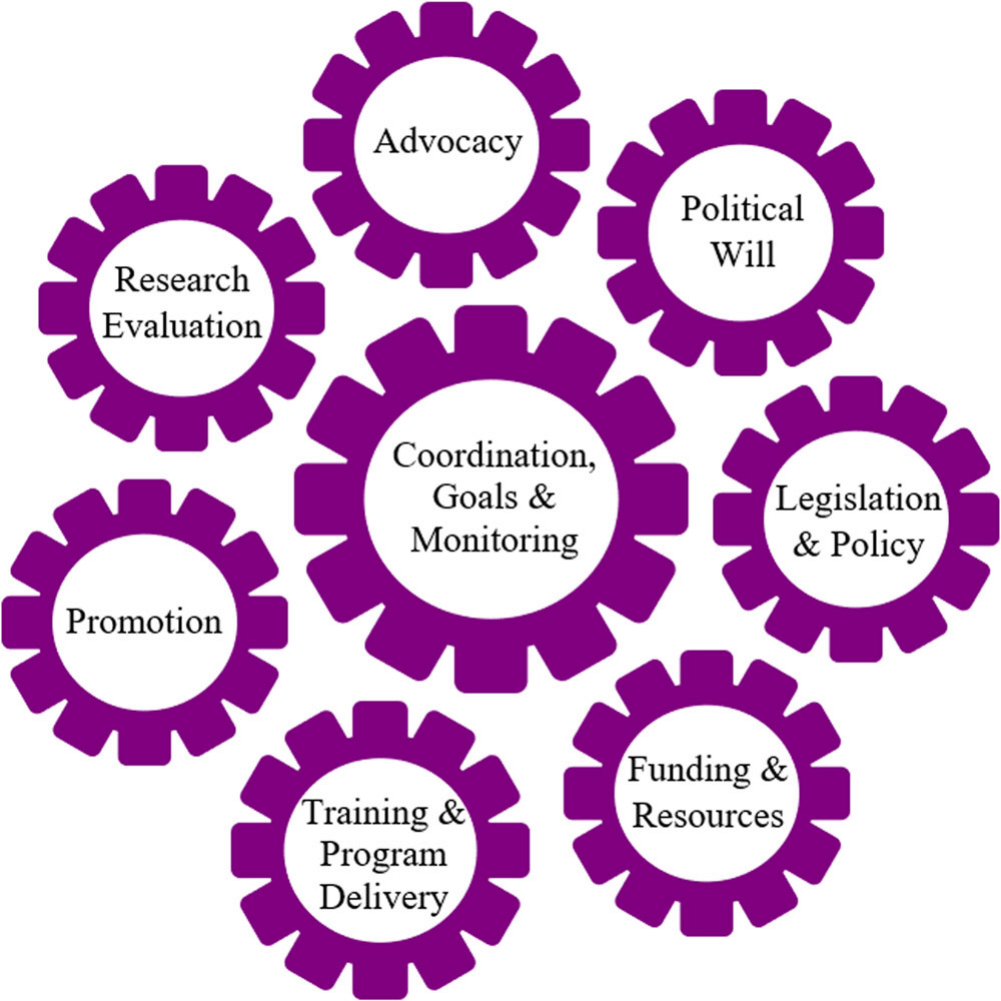
The BBF Gear Model details the BBF Gear Model, depicting how the gears are all interlinked and move one another to create a positive environment for change and one which is supportive of breastfeeding

BBF is grounded in the Breastfeeding Gear Model complex adaptive systems framework (Pérez‐Escamilla & Hall Moran, 2016). The Gear Model (see Figure 1) is made up of eight simultaneous conditions that sustain breastfeeding referred to as the ‘gears’ (Pérez‐Escamilla et al., 2012). This conceptual model illustrates how each gear must be sufficiently mobilised to turn the next, while the central Coordination gear gathers and delivers timely feedback. As depicted in Figure 1, in total there are eight interconnected gears. Those eight gears are:

1. Advocacy
2. Political will
3. Legislation and policies
4. Funding and resources
5. Training and programme delivery
6. Promotion
7. Research and evaluation
8. Coordination, goals and monitoring.

### 1.2 Scoring of the BBF gears

For each of the eight gears, there are a number of benchmarks. Each of the 54 BBF benchmarks are scored as follows: 0 (no progress), 1 (minimal progress), 2 (partial progress) or 3 (major progress). Each benchmark has specific criteria used to score. The country BBF committee must reach consensus on each score before it is recorded.

The eight gear scores show the strength of a country's current environment within each gear for scaling up breastfeeding protection, promotion and support programmes and initiatives. The eight gear scores are then used to calculate the final BBF Index Score: the strength of a country's current national enabling environment as a whole to scale up breastfeeding programmes and initiatives.

This paper focuses on the findings from the *Research and Evaluation gear* and the *Coordination, Goals and Monitoring gear*. These gears were selected to focus on in this paper as they were seen as high priority goals for England by the BBF committee due to the lack of available breastfeeding data at population level and the inconsistencies and gaps among the routine data in England. The lack of data makes it harder to undertake reliably consistent research and also to justify the need for greater funding. Further, the lack of the central strategic coordination and monitoring weakens oversight and the potential for system‐wide advances in the breastfeeding environment.

## 2 METHODS

A team of breastfeeding experts and key officials from Scotland, Wales and England attended the first BBF‐GB Engagement Committee in December 2017, agreeing to deliver BBF separately in each country to reflect structural and cultural variation. The three‐country committee was led by the University of Kent. The overall approach to the BBF methodology is described in Kendall et al., 2022 (BBF‐GB paper). This paper presents the BBF England process and findings. BBF work in Scotland and Wales are described respectively by McFadden et al. (2022) and Brown et al. (2022).

The process for the BBF work in England is presented below, detailing the steps that were realised.

### 2.1 Step #1: Establishment of the committee

The BBF England committee comprised key representatives from the Breastfeeding Network (BfN), the Department of Health and Social Care (DHSC), the Institute of Health Visiting (iHV), two professional colleges: the Royal College of Midwives and the Royal College of Paediatrics and Child Health (RCPCH), UNICEF UK Baby Friendly Initiative (BFI), Unite, University of Central Lancashire (FD was also on the Yale Technical Advisory Group for the BBF process) and the World Breastfeeding Trends Initiative (WBTi). It was co‐chaired and facilitated by the University of Kent, and Public Health England (PHE, now the Office for Health Improvement and Disparities).

### 2.2 Step#2: Evidence review process

**Figure 2:**
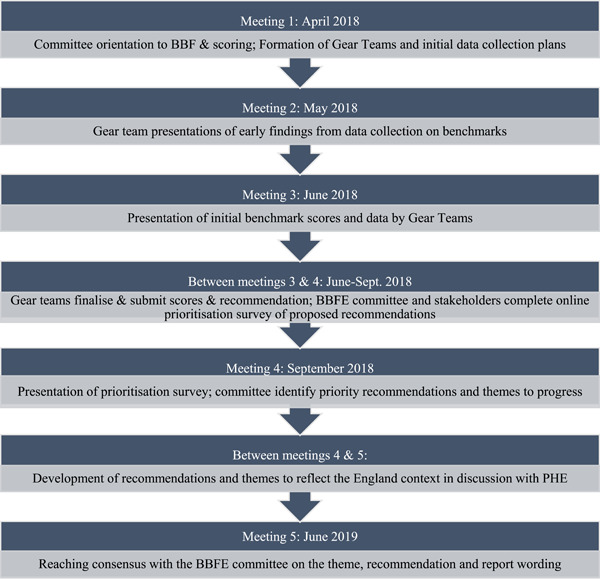
BBF meeting process for England lists the meeting process for BBF England. Five meetings were held in total over a 14‐month time period.

Following the standardised process developed by Yale University and using document and media searches, collaborative reviews and interviews, in April 2018 the BBF England committee started the process of gathering evidence from the previous 12 months and developing scores for England based on 54 benchmarks. Over the allocated period, the committee was required to deliver:

1. a series of evidence‐based scores for each benchmark within the gears, demonstrating areas of relative strength and weakness
2. a total gear score for each of the eight gears (a mean of the benchmark scores falling under that gear) providing an overview of the gear
3. an overall weighted BBF Index score for England, representing the strength of the scaling up environment. Some adaptations were made to the timeframe in response to the wider political context in England. These are detailed in Figure 2.

### 2.3 Step #3: Prioritisation of recommendations

**Figure 3:**
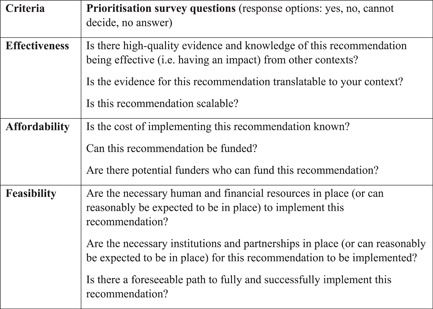
Recommendation prioritisation survey criteria and questions details the prioritisation survey questions asked of Becoming Breastfeeding Friendly (BBF) England committee members and stakeholders to grade and prioritise the initial BBF England recommendations.

After completing the data review for all the 54 benchmarks, each gear team made a set of recommendations targeting the gaps identified in the scoring process. The total set of 32 recommendations were then prioritised through an online survey. The process, adapted by Yale University for BBF purposes, is based on the Child Health and Nutrition Research Initiative (Rudan et al., 2008) research priority‐setting methodology. The survey was delivered by the University of Kent and asked respondents nine closed questions about the effectiveness, affordability and feasibility of each recommendation (see Figure 3). Each of the response options was allocated a score (‘yes’: 1, ‘cannot decide’: 0.5, ‘no’: 0, ‘no answer’: blank). The survey link was circulated to all BBF England committee members, as well as BBF‐GB members and nominated relevant individuals. The process generated scores representing a range of perspectives for each recommendation's effectiveness, affordability and feasibility, as well as an overall mean score indicating the respondents' overall level of support for—and therefore prioritisation of—each recommendation.

Figure 3 details the prioritisation survey questions asked of BBF England committee members and stakeholders to grade and prioritise the initial BBF England recommendations.

In September 2018, the BBF England committee considered the prioritisation survey findings, noting the overlap among the recommendations and the emerging thematic areas. For example, 8 of the 32 recommendations called for the development of a strategic oversight group, and a further 5 referred to strengthening strategic planning and oversight mechanisms. In response, six recommendations were made drawing on the highly prioritised areas to achieve impact in the strengthening of the breastfeeding environment for women, babies and families and progress breastfeeding rates in England towards national and global targets. The recommendations were further reviewed by Public Health England and DHSC colleagues in light of the England context, in consultation with the University of Kent. The wider BBF England committee came together for the final meeting in June 2019 to reach consensus on the theme, recommendations and report wording.

### 2.4 Scoring methods for the Research and Evaluation and the coordination, goals and monitoring gears

#### 2.4.1 The Research and Evaluation gear

The Research and Evaluation gear assesses the (a) availability, integration and monitoring of key breastfeeding practices; and (b) availability of monitoring systems to track implementation of activities essential to the scaling up of breastfeeding. The gear includes ten benchmarks focused around two themes:

1. Breastfeeding outcomes (benchmarks 1–5)
2. Monitoring process indicators (benchmarks 6–10).

**Figure 4:**
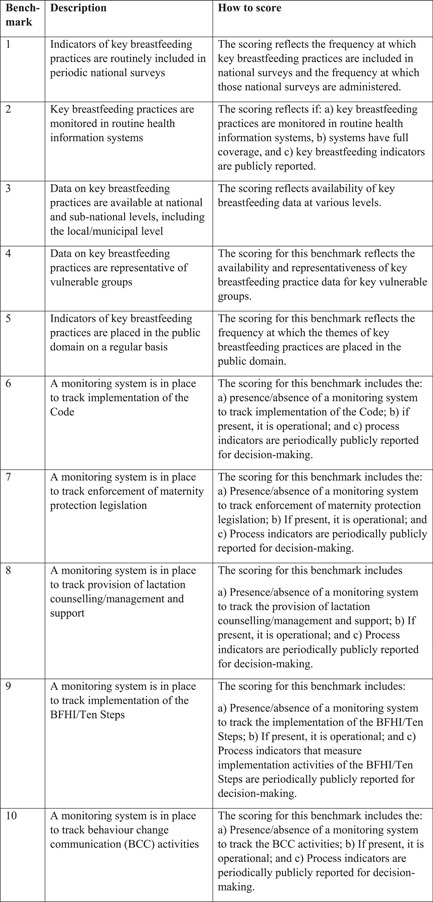
Research and evaluation gear benchmarks explains how the scoring was done for the Research and Evaluation gears. Everyone involved in the BBF committee were given the details on how to score and discussed them in advance, before starting the review of the evidence. *Source*: Yale Public Health. Research and Evaluation < Becoming Breastfeeding Friendly: A Guide to Global Scale Up (yale. edu).

Figure 4 details the benchmarks and the scoring process.

#### 2.4.2 The coordination, goals and monitoring gear

**Figure 5:**
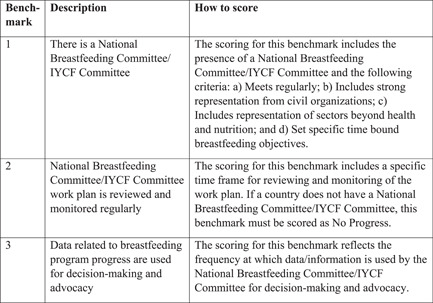
Coordination, goals and monitoring gear benchmarks. *Source*: Yale Public Health. Coordination, Goals and Monitoring < Becoming Breastfeeding Friendly: A Guide to Global Scale Up (yale. edu) explains how the scoring was done for the coordination, goals and monitoring gears. Everyone involved in the BBF England committee were given the details on how to score and discussed them in advance, before starting the review of the evidence.

This gear explores if there is a government system responsible for coordinating the breastfeeding programme at a national level and, if operational, whether it allows for effective decision making from the national to the local level. In this respect, this gear serves as the master gear, which sets and monitors overall goals and ensures all gears receive timely feedback, thereby enabling the breastfeeding programme machine to function properly. The gear includes three benchmarks focused on goal setting and feedback. Figure 5 details the benchmarks and the scoring process.

## 3 RESULTS

**Figure 6:**
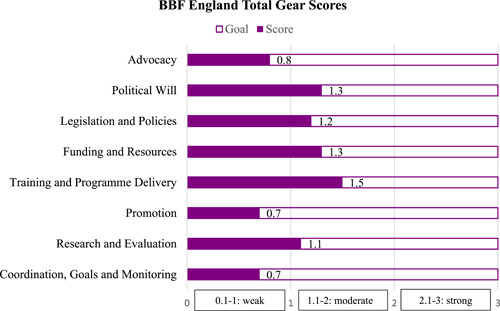
Overview of the gear scores for England details the scores given to each of the Gears by the BBF England committee. The committee was made up of many individuals from the NHS, government, nongovernment, public health, professional and academic organisations.

England's overall weighted BBF Index score was 1.1 from a possible range of 0–3, representing a moderate scaling up environment (1.1–2.0). Figure 6 illustrates the total gear scores for England. Five gears: *Political Will, Legislation and Policies, Funding and Resources; Training and Programme Delivery and Research and Evaluation* scored at a moderate gear strength, while *Advocacy, Promotion and Coordination Goals and Monitoring* were weak.

The Training and Programme Delivery gear received the highest score due to the fact that learning outcomes do exist for health professionals and practitioners, for peer supporters and for specialist services, though there is a need for greater coordination and integration. Some consistency is provided however in training for midwives and health visitors through UNICEF UK BFI accreditation, though coverage is not universal. The lowest scores were given for Promotion and Coordination, Goals and Monitoring. A low score of 0.8 was concluded for promotion due to the lack of a dedicated national strategy for breastfeeding and for promotion in particular, alongside a singular authority for direction, oversight, evaluation, securing resource, reviewing effectiveness and establishing efficacy in relation to the England contexts and societal drivers. While local promotional strategies and programmes exist, there is a lack of data collection, sharing and learning.

### 3.1 Research and Evaluation gear

The Research and Evaluation gear scored a mean total of 1.1, from a range of 0–3. The review of the evidence highlighted a number of key gaps which contributed to the scoring given.

#### 3.1.1 Breastfeeding outcomes (benchmarks 1–5)

Up until 2010, the UK had a national Infant Feeding Survey which had been conducted every 5 years since 1975. Since that was cancelled, no population level data has been collected specific to infant feeding. In England breastfeeding practice data has been collected at the 6–8 week postnatal check‐up with mothers (Office for Health Disparities and Improvement Fingertips public health data, 2022). This data is routinely collected, and reports are updated annually and placed in the public domain. The data is published to local level, allowing for comparisons across regions where sufficient data has been submitted. The data set can be cut in a range of ways, including by age of mother, ethnicity, postcode and so forth. however there is some concern that the data sets do not adequately identify or represent vulnerable groups (Aspinall, 2014). The developing CSDS records personal patient data and risk indicators such as ‘social and personal circumstances’ or ‘safeguarding vulnerability factors’ (relating to the child as opposed to the mother) (NHS Data Model and Dictionary, 2021). The CSDS is not currently in the public domain, though it may be accessed through NHS Digital to access ‘research ready’ subsets of data (Fraser et al., 2020). Concerns persist about the accuracy of the data due to potential miscoding or misclassification by health professionals entering the data (Fraser et al., 2020).

However, the BBF evidence review found a lack of longer‐term breastfeeding practice data being collected, making international comparisons unfeasible. The quality and accuracy of the data across the different local areas is also an issue. As a result, the BBF process for England delivered strong evidence for more robust routine infant feeding data collection and reporting. This would require systems to include recognised time‐points up to 2 years of age to better inform infant feeding monitoring and action planning and delivery at national, regional and local levels. Further, noting the cessation of the national infant feeding survey, the BBF committee delivered strong evidence on the value of consistent population level data gathering through a survey of infant feeding, conducted at set (5 year) intervals, that focuses on key data, the parent experience and building internationally comparable data.

#### 3.1.2 Monitoring process indicators (benchmarks 6–10)

The UK's legislation, named 'The Infant Formula and Follow‐On Formula Regulations’, incorporates some of the International Code of Marketing of Breast Milk Substitutes into law^1^. Despite this, the BBF committee found that there was no capacity to enforce the law. It was also discovered that while companies are obliged to share information on new projects with the DHSC, the Department is not obliged to share this information with other organisations involved in the monitoring of infant formula companies. This finding highlighted a lack of transparency across the system.

In relation to the implementation of the UNICEF UK BFI standards, there is a monitoring system in place across accredited sites and services. However, the UNICEF UK BFI programme at the time of the scoring was voluntary in England and deemed potentially less stable as a result. The WHO has previously expressed concern that so many monitoring systems are voluntary and therefore vulnerable (World Health Organization, 2018). The committee found that PHE do track their behaviour change communication activities. However, this was for internal use and not done routinely. Local level monitoring and reporting is also delivered internally and used predominantly to justify funding outlay.

### 3.2 Coordination, goals and monitoring gear results

This gear scored a mean total of 0.7, from a range of 0–3. A number of issues and gaps were identified which led to the low score and weak gear strength rating. England has no National Breastfeeding Committee or Infant and Young Child Feeding Committee that meets the specified criteria for the gear benchmarks. This is in respect to there being no committee which (a) Meets regularly; (b) Includes strong representation from civil organizations; (c) Includes representation of sectors beyond health and nutrition; and (d) Sets specific time‐bound breastfeeding objectives. Although from 2008 to 2011 there was a specific national infant feeding coordinator role at the Department of Health England and nine part‐time employed regional coordinator positions, since 2011 there has been no national coordinator.

This lack of a national infant feeding coordination role or national breastfeeding committee has resulted in no specific workplan for breastfeeding being developed. However, the Improving Prevention and Population Health Workstream 9 of the Maternity Transformation Programme (NHS England, 2018) does have a priority in it to increase the number of babies breastfed at 6 months. There was also an identified high impact area for breastfeeding under the Healthy Child Programme that was updated in 2021, post the BBF process (https://www.gov.uk/government/publications/commissioning-of-public-health-services-for-children/early-years-high-impact-area-3-supporting-breastfeeding). However, the problems of data collection and monitoring remain.

Finally, the BBF committee members concluded that although data does inform some decision making and advocacy in breastfeeding programmes, there was a lack of cohesive strategy and no single authority had oversight or coordinated the response to low breastfeeding rates. As a result of this evidence, the BBF process for England identified the need for greater coordination, strategic goal setting and consistent monitoring.

### 3.3 Recommendations

**Figure 7:**
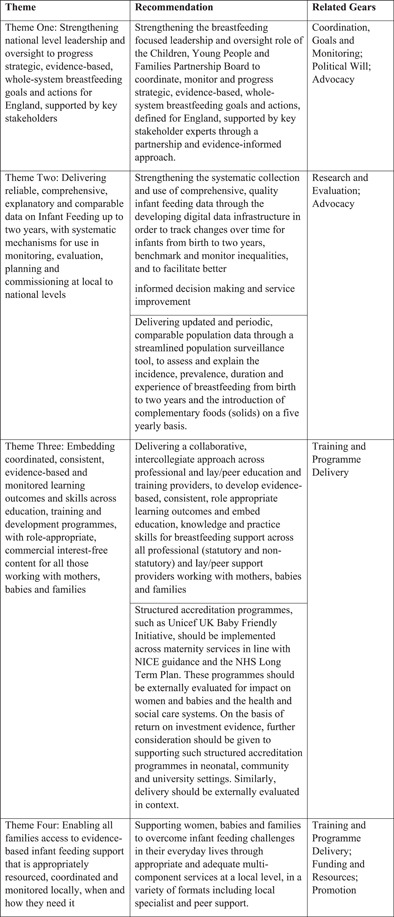
Themes and final recommendations corresponding to BBF gears details the final recommendations made, by gear

Across all the gears, 32 data driven and evidence‐based recommendations were made and then prioritised through an online survey (described above), two BBF England committee meetings and virtual negotiation to achieve consensus across members. This process resulted in six recommendations, two focused on Research and Evaluation, and one on coordination, goals and monitoring. The three remaining recommendations focused on findings from other gears (Figure 7).

The initial set of pre‐prioritisation recommendations made specifically for the Research and Evaluation gear and the Coordination, Goals and Monitoring gear are detailed below.

#### 3.3.1 Research and Evaluation gear: Recommendations

The following seven initial recommendations were made:

1. A comprehensive, tailored Infant Feeding Survey is conducted at (ideally) 5‐year intervals, with a maximum 10‐year interval.
2. Routine breastfeeding data collection is refined and supplemented to better reflect and plan for existing and evolving vulnerable groups.
3. Data collection in the routine datasets is developed to include key breastfeeding practices beyond 6–8 weeks to include breastfeeding at 6 months, 1 year and 2 years and duration of breastfeeding.
4. The collection of good quality data is improved through a more meaningful connection with its use for strategic and operational decision making.
5. An Infant and Young Child Feeding Strategy (IYCFS) is developed for England, guided by a multi‐agency and multi‐disciplinary IYCFS Board; a Monitoring Strategy is codeveloped and incorporated as a central element.
6. A primary function of the IYCF Monitoring Strategy will be to develop structured, funded monitoring mechanisms, tailored to ensure sustainable, consistent monitoring of programmes, provision and violations/enforcement of relevant legislation by named agencies/groups.
7. Full implementation of the Unicef UK BFI across maternity, community and neonatal services is mainstreamed with sufficient resourcing as a strategic approach to extending local BFI monitoring to all maternity, community and neonatal settings.

The recommendations for improved data collection and the introduction of an Infant Feeding Survey, are similar to recommendations made by four other UK‐based organisations in the past few years, including the Scientific Advisory Committee on Nutrition (2018), World Breastfeeding Trends initiative (2016), Royal College of Paediatrics and Child Health (2017), and UNICEF UK Baby Friendly Initiative (2017). The need to collect more routine data on breastfeeding practices was also linked to the National Institute of Clinical Excellence (NICE) Guidance CG110 (National Institute of Clinical Excellence NICE, 2010) which refers to the development of models for service provision for women with complex social factors, based on the evidence. More refined data is necessary for greater understanding of trends, breastfeeding behaviour and critical points, such as drop off and cessation among vulnerable groups. Such data would help in planning and operational, as well as strategic decision‐making.

#### 3.3.2 Coordination, goals and monitoring gear: Recommendations

Three initial recommendations were made by the Coordination, Goals and Monitoring gear team.

1. Establish an overarching Infant Feeding Committee with the responsibility for managing the interface with national and local government.
2. Developing standards for promotion, use of valid evidence, advocacy, funding, monitoring.
3. Supporting the monitoring of compliance with existing legislation and the international code and influences educational standards for the professions and supporters.

Other gear teams put forward a total of 10 further recommendations relating to the need for greater coordination, goal setting and monitoring to strengthen their gears. The additional recommendations supported and extended those made above with the following points: the multi‐disciplinary infant feeding group should have sufficient knowledge, skills, power and influence to drive strategy; to ensure consistent monitoring and oversight; and to codevelop standards underpinned by the evidence.

The recommendation for an overarching Infant Feeding Committee which would act as a conduit and expert repository for the media and enable co‐ordination of activities was in part informed by Brazil's National Breastfeeding Committee (CNAM) (Yale School of Public Health, 2018). The Committee was reformulated to include representation from multiple sectors including government, universities and civil organisations. CNAM is regarded as a powerful group that uses evidence to strongly advocate for breastfeeding as a government health priority and supports their Ministry of Health in decision making (Yale School of Public Health, 2018). The many examples of the committee's success include legislation to refine the BFHI criteria and the WBTI scores for National Policy, Programme and Coordination have been 9.5 and 10 out of 10 for 2008 and 2014, respectively (Yale School of Public Health, 2018). The BBF England committee felt that this evidence indicated that a properly functioning, funded, implemented and coordinated National Committee could be highly effective.

### 3.4 Developments since the BBF processes concluded

The BBF England recommendations were presented to Public Health England in July 2019. While the findings were well received there have been systemic delays in bringing the recommendations into full action and progress. This has been due a complex political environment involving the Brexit agreement and a General Election in 2019, followed by the outbreak of the COVID‐19 pandemic in 2020. However, in the interim the Prevention Green Paper was published by the UK Government which makes a clear commitment to promotion of good health, prevention of ill health and reduction of health inequalities through a personalised approach to prevention. Clearly, the BBF England evidence points to breastfeeding forming part of this agenda, and the PHE team have been in touch with the authors explaining that discussions internally are still on‐going, and they plan to make changes in line with some of the recommendations in the near future.

In addition, there is commitment to promotion of breastfeeding with additional funding for community support and the implementation of the Unicef UK Baby Friendly standards across services, through the maternity services as part of the Best Start for Life programme (HM Government, 2021) and a commitment to modernising the Healthy Child Programme (Public Health England, 2021) to which the BBF England findings have been presented. It remains a clear gap that the key recommendations discussed in this paper have not yet been fully accepted or implemented, presenting a challenge for policy implementation research in the public health arena. England's positive experience with BBF supports findings from other countries with this initiative including Germany (Flothkötter et al., 2018), Ghana (Aryeetey et al., 2018), Mexico (González de Cosío et al., 2018), Myanmar (Than et al., 2019), Samoa (Soti‐Ulberg et al., 2020), Scotland (McFadden et al., 2022) and Wales (Brown et al., 2021). Thus, while the authors are aware that some more recent changes are underway in England, there continues to be a need for further policy research in relation to implementation science that will contribute to elucidation of the tensions between research, policy and the promotion, protection and support of breastfeeding in England.

## 4 DISCUSSION

The BBF process for England identified the need for strategic goal setting. Such goal setting is necessary as resources are finite (Robinson et al., 2012). However, the process of priority‐setting is inherently political, and is a multi‐faceted process which is informed by more than the evidence base. Although the evidence analysis should feed into the formulation of strategic goal setting, the reality is that strategic goal setting is a process which is influenced by many stakeholders and other factors, including lobbyists, public opinion, traditions and social norms, as well as the evidence‐base (Mitton et al., 2009; Terwindt et al., 2016).

To set and monitor any strategic goal, there needs to be consistent and meaningful data across all geographical areas in England collected. This data needs to examine both the numbers and the experience of infant feeding for mothers, babies and families at several key points during the first 2 years of life. Without routinely collected, quality breastfeeding data, countries lack the ability to comprehensively monitor their progress. The UK Infant Feeding Survey was conducted every 5 years between 1975 and 2010. The main aim of the survey was to provide estimates on the incidence, prevalence and duration of breastfeeding and other feeding practices adopted by mothers in the first 8–10 months after their baby was born, and this data is now largely missing. The Office for Health Improvement and Disparity (previously Public Health England) collect breastfeeding data for the first feed and at 6–8 weeks but this does not provide a full analysis, there is no nationally available data on exclusive breastfeeding at 6 months for example. England is not alone in its lack of data around breastfeeding; it is reported that only 40% of countries have data on exclusive breastfeeding from the last 5 years. However, England's inability to track funding for breastfeeding programmes or identify how much of the national budget is allocated to support breastfeeding interventions is of concern (Global Breastfeeding Collective, 2019), especially considering the fact that breastfeeding has been defined as the most cost‐effective public health intervention (Molbak et al., 1994; World Health Organisation, 2000).

The BBF England process and review of the evidence also highlights the need for a strengthening of the relevant breastmilk substitute marketing legislation, combined with the appropriate monitoring and action on violations of this legislation. Through clever marketing the formula milk industry has managed to position their products as a women's right to choose, and that by proposing exclusive breastfeeding, governments and health boards are somehow taking women's choices away and stigmatising those women who choose not to breastfeed (Hastings et al., 2020). Governments need to go beyond simply promoting breastfeeding as a ‘good thing’; they need to create supportive policies and programmes to enable the environments that parents need such as maternity benefits, regulation of marketing practices from the infant formula companies that go against the WHO Code, breastfeeding facilities in public spaces, desexualizing of the breastfeeding body and the experiences of mothers that find breastfeeding difficult.

The need for greater coordination was a final conclusion from the BBF England study. BBF's evidence based Breastfeeding Gear Model advocates central coordination to ensure multi‐sectoral public health programmes remain on track through setting and monitoring goals, facilitating the flow of information across gears and providing timely feedback on actions needed to improve or sustain the quality of scaled up programmes. A cross sectional survey of practitioners from predominantly industrialised countries (Rosin & Zakarija‐Grković, 2016) suggests the following enablers for the impact of national breastfeeding coordination on breastfeeding rates: being empowered and supported to deliver national leadership by their governments; working transparently to strengthen strategy and policy; and ensuring appropriate funds, power and influence.

## 5 CONCLUSION

The BBF England process highlighted clear gaps within the current breastfeeding evidence, policy, and approach in England—and generated six final recommendations. In this paper, we focus on the need to strengthen infant feeding coordination and strategic action through an overarching national committee and improve data collection through robust collection mechanisms which record and track infant feeding data across the first 2 years of life as well as parent voices and local spending. However, while the recommendations were presented, prioritised and agreed upon in 2019, the response to the recommendations and action by government has been slow to progress affected by the complicated political environment in England with the Brexit agreement and a General Election in 2019, followed by the outbreak of the COVID‐19 pandemic in 2020. Despite this, in 2022 the Best Start for Life programme for the First 1001 Critical Days (HM Government, 2021) led by Andrea Leadsom has resulted in further funding for community support for breastfeeding. With the recent commitment provided by the new British Prime Minister for the Early Years Agenda (Leadsom, 2022), we look forward to a refocus and action on the BBF recommendations for England. It will be an important next step to undertake a further round of the BBF process to evaluate changes and improvements in England since 2019.

## AUTHOR CONTRIBUTIONS

Sally Kendall designed the study based on the Yale protocol developed under the leadership of Rafael Pérez‐Escamilla; Rowena Merritt, Sally Kendall and Tamsyn Eida collected, analysed and interpreted the data with the BBF England committee members; Rowena Merritt drafted the manuscript; all authors revised the manuscript critically for important intellectual content and approved the final version.

## CONFLICT OF INTEREST

The authors declare no conflict of interest.

## Data Availability

The data that support the findings of this study are available from the corresponding author upon reasonable request.

## Footnotes

1 These regulations cover infant formula intended for babies under 6 months old; they do not cover any food, and do not cover the products for babies older than 6 months. Source: UNICEF: The International Code of Marketing of Breastmilk Substitutes—Baby Friendly Initiative (unicef.org.uk).
